# Optimal Patient Positioning for Microvascular Decompression of Trigeminal Neuralgia Utilizing the “Arrowhead” Technique: A Technical Report

**DOI:** 10.7759/cureus.75966

**Published:** 2024-12-18

**Authors:** Tyler E Rice-Canetto, Louis Reier, Mohammad Arshad, Michael Schiraldi, Javed Siddiqi

**Affiliations:** 1 Neurosurgery, Arrowhead Regional Medical Center, Colton, USA; 2 Neurosurgery, California University of Science and Medicine, Colton, USA; 3 Neurosurgery, Riverside University Health System Medical Center, Moreno Valley, USA; 4 Neurosurgery, Desert Regional Medical Center, Palm Springs, USA; 5 Neurosurgery, Redlands Community Hospital, Redlands, USA

**Keywords:** management of trigeminal neuralgia, microvascular decompression (mvd), microvascular decompression surgery, neurosurgical approaches, neurosurgical techniques, park bench, park bench positioning, patient position, surgical position, trigeminal neuralgia

## Abstract

Microvascular decompression (MVD) is a neurosurgical operation used to treat trigeminal neuralgia (TN). The surgery is performed through a retrosigmoid approach, where a Teflon pledget is placed in between the offending vessel (most commonly the superior cerebellar artery) and trigeminal nerve. The surgery is performed within the superior aspect of the cerebellopontine angle (CPA) through a small working corridor that is triangulated by the petrous bone and tentorium. Patient positioning is the foundation to accessing this corridor and performing a successful operation. This article provides a detailed guide on the positioning steps of the park bench position typically employed at Arrowhead Regional Medical Center (ARMC) in Colton, CA, USA. We then review pertinent surgical anatomy in a MVD for trigeminal neuralgia and describe the “Arrowhead" technique to perform this operation.

## Introduction

The park bench position, or three-quarters prone position, is a modified lateral decubitus position used in neurosurgery for accessing the posterior fossa. This position offers excellent exposure to the cerebellopontine angle (CPA) with minimal brain retraction, making it the ideal position to perform a microvascular decompression (MVD) for trigeminal neuralgia (TN). Understanding the nuances of positioning is critical not only for accessing the correct surgical corridor but also for preventing complications such as nerve injury, pressure ulcers, and compromised respiratory function [1]. The park bench position can be cumbersome, time-consuming, and energy-depleting if not well-versed in the positioning steps. This report provides a detailed step-by-step guide on positioning the neurosurgical patient in the park bench position for a MVD of TN using the “Arrowhead" technique.

This technical report shares many similarities with the article published by Marotta et al., titled “Perioperative Positioning in Neurosurgery: A Technical Note on Park Bench Positioning for the Obese Patient Using the ‘Arrowhead" Technique" [1]. However, there are several differences that make this article unique. While the report by Marotta et al. highlights the nuances of the park bench position for the obese patient, this article applies to all patient populations and places a greater emphasis on how to perform the correct order of steps to ensure positioning is performed appropriately, safely, and expeditiously. Further, we review relevant surgical anatomy in a MVD for TN and describe the Arrowhead technique for performing other key aspects of this operation.

## Technical report

Part I: the Arrowhead technique, park bench positioning

Step 1: Prepositioning Preparation

Prior to the patient arriving in the operating room, it is imperative to have all supplies that will be necessary for patient positioning. The supplies required include the following: axillary roll, egg crate foam paddings, lower arm support (multi-position arm board with clamp), Mayfield apparatus, pillows, surgical bean bag positioner, draw sheet, 3-inch silk tape, and three Velcro straps [1]. For obese patients, the Krause-style arm support (with a properly fitted stocking) should also be obtained [1]. The draw sheet should be placed horizontally across the operating table with the upper end of the draw sheet positioned approximately 12 inches below the head of the table. The surgical bean bag is then positioned centrally on top of the draw sheet. The three Velcro straps are hung from the operating table side rails [1].

Intubation, induction of general anesthesia, and appropriate arterial and intravenous lines are placed prior to transferring the patient onto the operating table. Additionally, it’s important to ensure proper padding at potential pressure points (including the knees, ankles, elbows, and hips).

Step 2: Initial Patient Transfer 

The patient is then transferred onto the operating table in a supine position, and the bed can be rotated 90 or 180 degrees away from anesthesia. Ensure the upper edge of the surgical bean bag positioner is approximately midway between the patient's sternal notch and xiphoid process (ideally, at the level of the nipples) [1]. The draw sheet used to transfer the patient should be retained for future positioning adjustments [1].

Step 3: Body Positioning

Lateral decubitus setup: Turn the patient into the lateral decubitus position so that the surgical site is face up and the patient is faced away from the surgeon. The bottom hip should be placed at the center of the table. 

**Figure 1:**
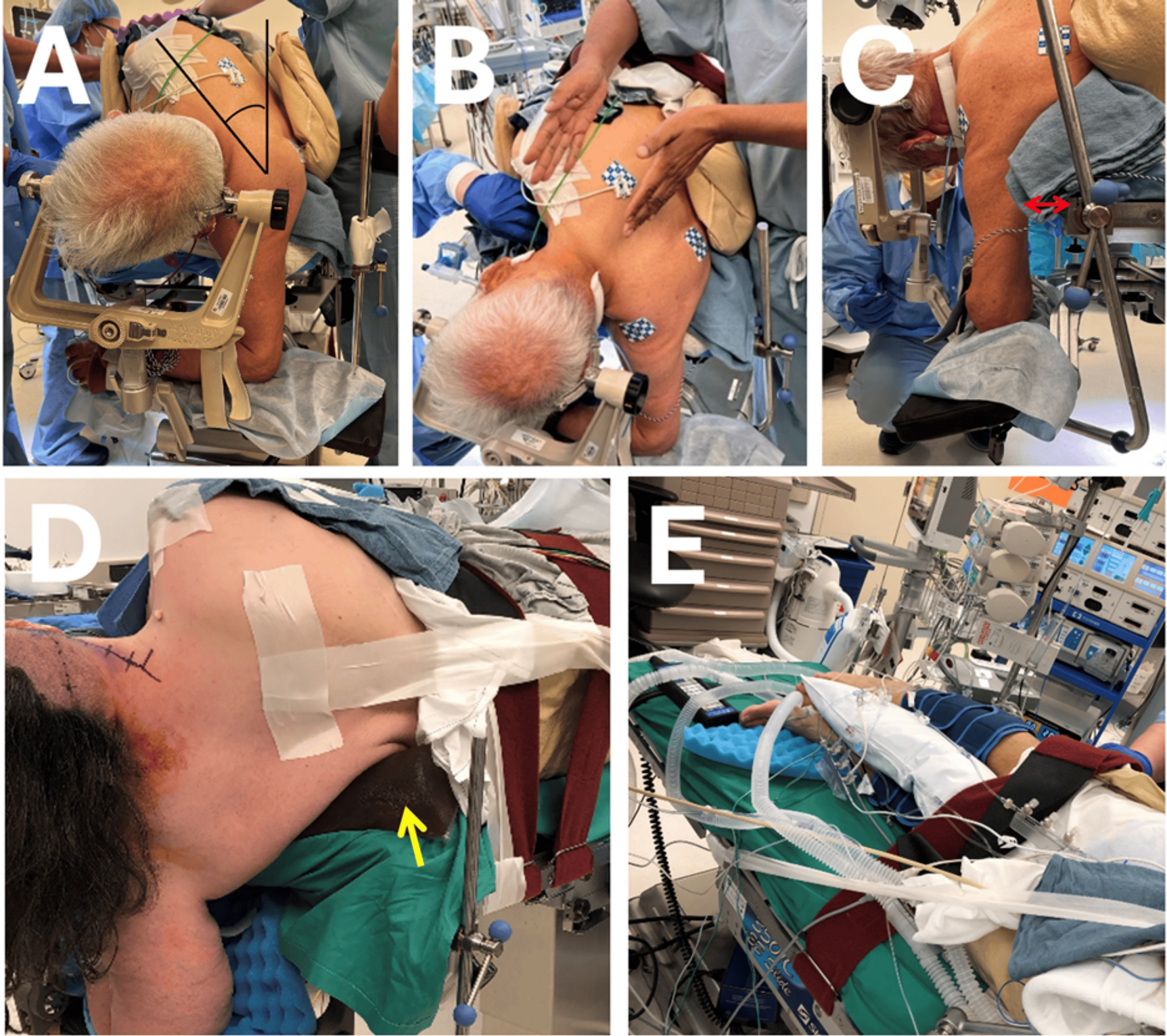
Body positioning A. The upper shoulder is turned 30-45 degrees away from the physician. B. The surgeon's body is ideally positioned for the operation. The surgeon's hands are referencing his line of sight and trajectory to the right upper cerebellopontine angle (CPA). C. The axilla is at least two fingerbreadths (double red arrow) past the edge of the table. D. The axillary roll (yellow arrow) keeps the axilla hanging freely and not compressed against the operating room (OR) table [1]. E. A pillow is placed between the legs [1].

Shoulder and hip alignment: The upper shoulder should be rotated forward approximately 30-45 degrees [2], as shown in Figure 1A. This optimizes the surgeon’s working space and trajectory to the superior lateral CPA (Figure 1B). Silk tape can be used to translate the shoulder anteriorly away from the surgical working space. It is this positioning of the shoulder and hips that differentiates the three-quarter prone position from the lateral decubitus position, as the patient appears to be “falling forward” given the hip and shoulder alignment in this position. 

Axilla: It should be at least two fingerbreadths past the edge of the table as shown in Figure 1C. An axillary roll is positioned under the upper thorax (not in the axilla) to ensure the axilla is kept free and not wedged against the operating table (Figure 1D). This maneuver prevents injury to the brachial plexus, arm ischemia, and compartment syndrome [3].

Legs: They are bent by slightly flexing the hips and knees. A pillow is placed in-between the legs as shown in Figure 1E.

Figures 1A-1E provide a visual demonstration of the surgical positioning of each of the aforementioned body parts.

Step 4: Securing the Body

**Figure 2:**
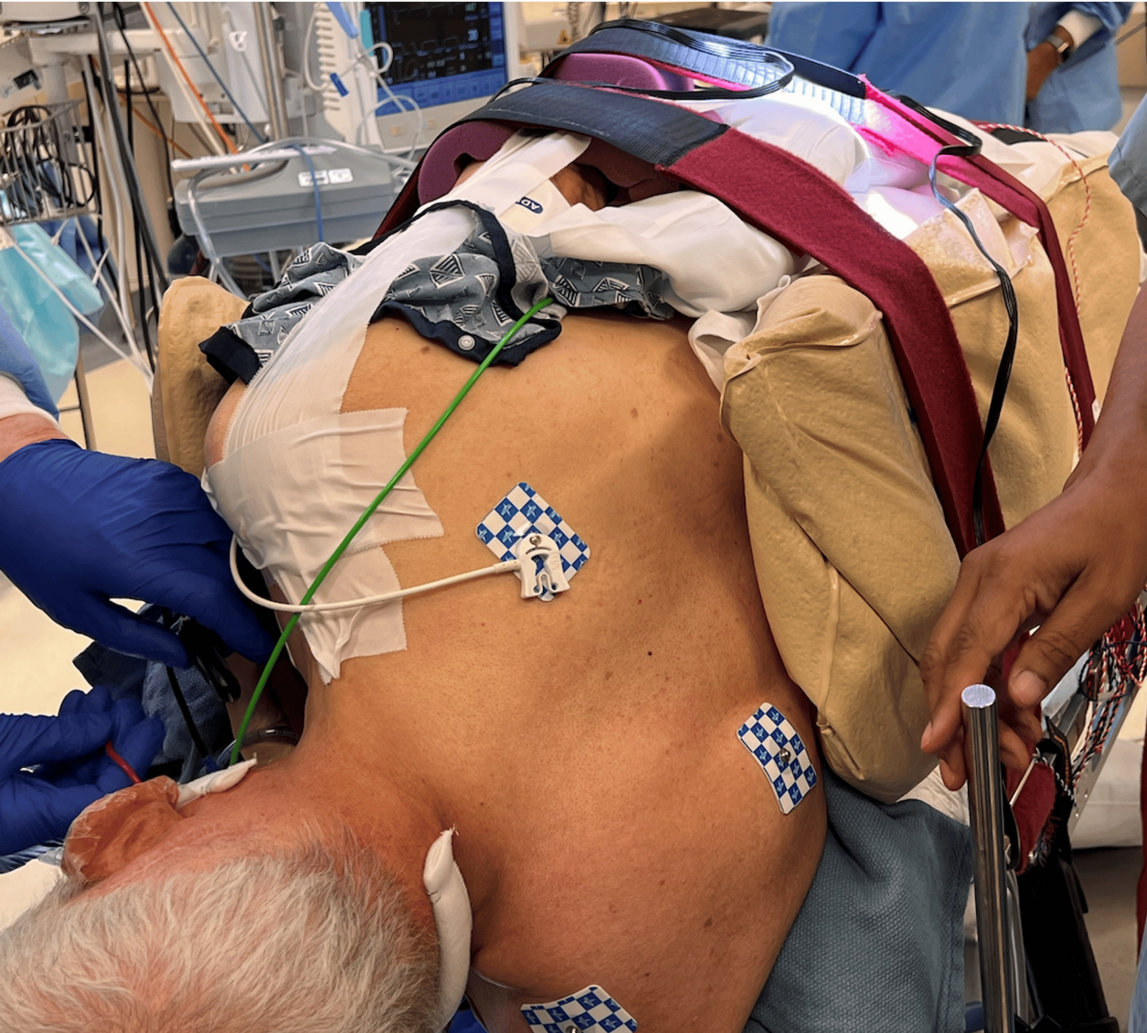
Bean bag stabilizer is inflated and conformed to the patient’s body.

Bean bag positioner: Once ideally conformed to the patient, apply suction, which will cause the bean bag to harden and stabilize the patient's body, as shown in Figure 2. Before setting the bean bag, ensure there aren’t any cardiac leads or tubing in direct contact with the patient’s body that will be compressed by the bean bag.

**Figure 3:**
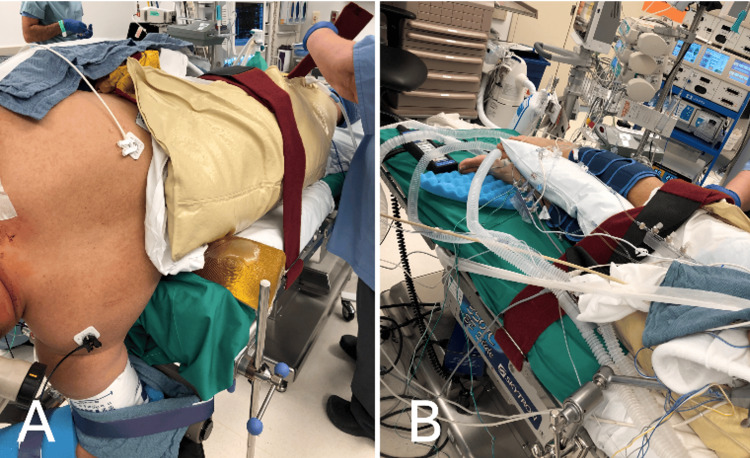
Perioperative images demonstrating proper strap placement A. Velcro straps are placed above the hips [1]. B. Velcro straps are placed above the knees and on the legs. A pillow is placed between the legs to avoid bony contact at the level of the ankles and knees [1].

Velcro straps: They are secured above the hips, above the knees, and on the legs as shown in Figure 3. Ensure that the straps do not cross joints, wounds, or vascular access points [1].

Step 5: Head Positioning and Fixation

**Figure 4:**
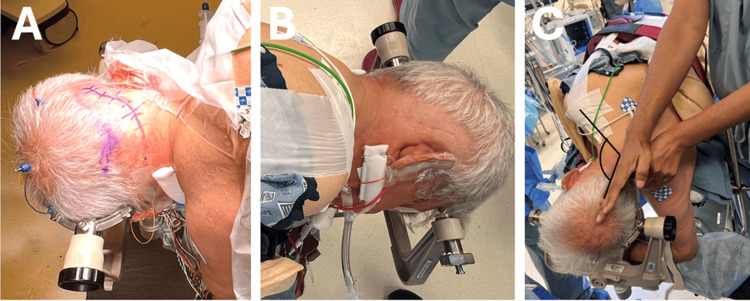
Head positioning and the Mayfield attachment A. The double pins are placed in the parietooccipital region on the side contralateral to the lesion. B. The single pin is placed in the frontal region, above and lateral to the lateral canthus, just below the hairline, on the side ipsilateral to the lesion. C. The vertex of the skull is positioned slightly down toward the floor, which creates a greater angle between the patient’s shoulder and neck. This gives the surgeon a larger working zone and provides the surgeon with the ideal line of sight to the superior-lateral cerebellopontine angle (CPA).

Up until this step, the patient’s head is held manually to ensure the head and neck are not moved in non-anatomical positions. Once the body is positioned, the Mayfield skull clamp can be attached to the patient’s head as shown in Figures 4A-4B. After verifying that the skull clamp is secure, the patient's head is positioned to optimize the surgical corridor (Figure 4C). The vertex of the skull is pointing down toward the floor. Ensure that two-finger breaths can fit between the chin and sternum to prevent jugular vein compression and venous congestion. Once the optimal trajectory is established, the Mayfield is secured to the operating table. 

Step 6: Arm Positioning

**Figure 5:**
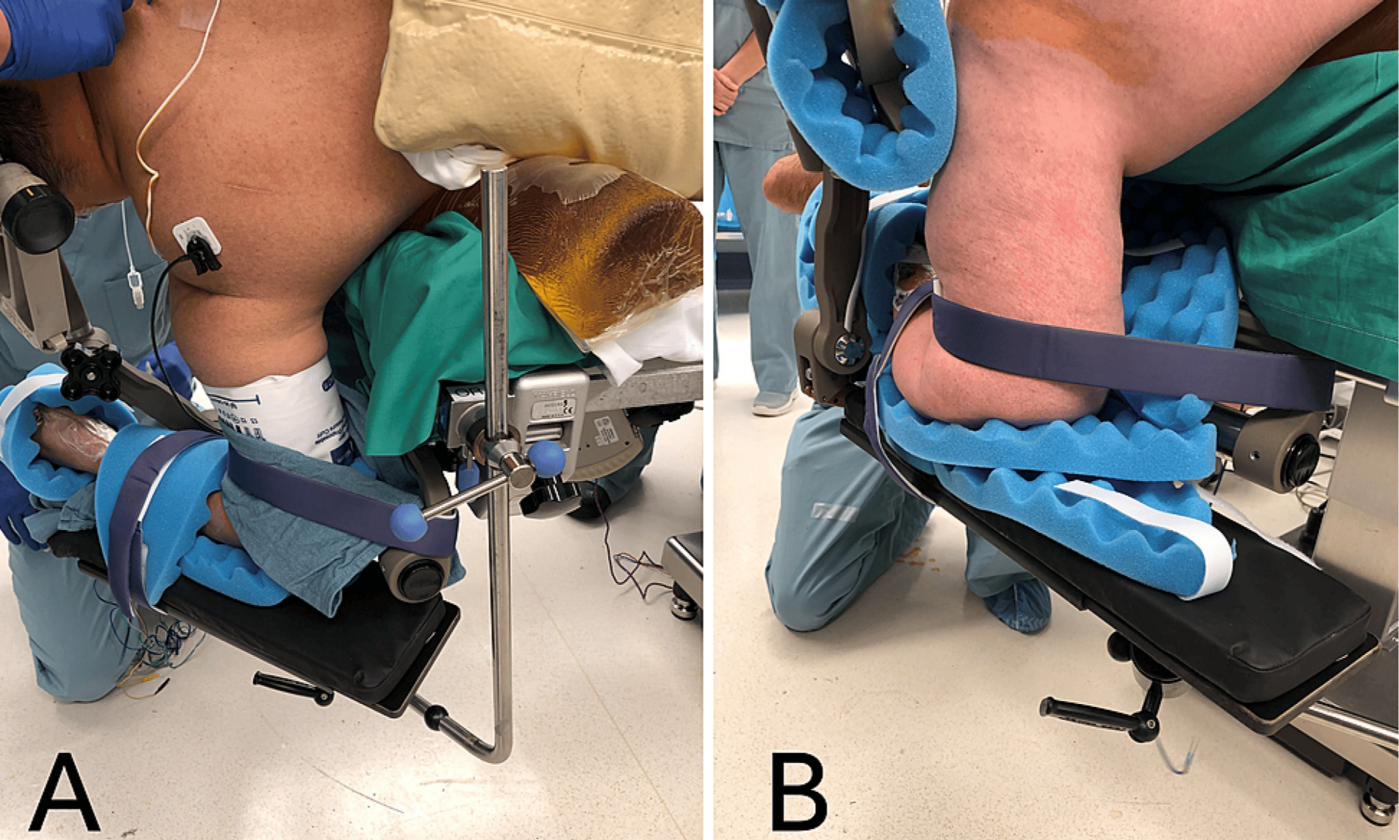
Lower arm positioning A. The arm board is positioned outside the Mayfield [1]. B. The arm board is positioned inside the Mayfield [1].

Lower arm: Secure the arm closest to the floor (dependent arm) using a multi-position arm board as shown in Figure 5. The arm board can be positioned above or below the Mayfield clamp depending on the patient's humerus length and glenohumeral joint laxity [1]. Figure 5A is an example of the arm board positioned outside the Mayfield, whereas Figure 5B is an example of the arm board positioned inside the Mayfield. Ensure that the wrist and elbow rest comfortably on the arm board without excessive pressure [1].

**Figure 6:**
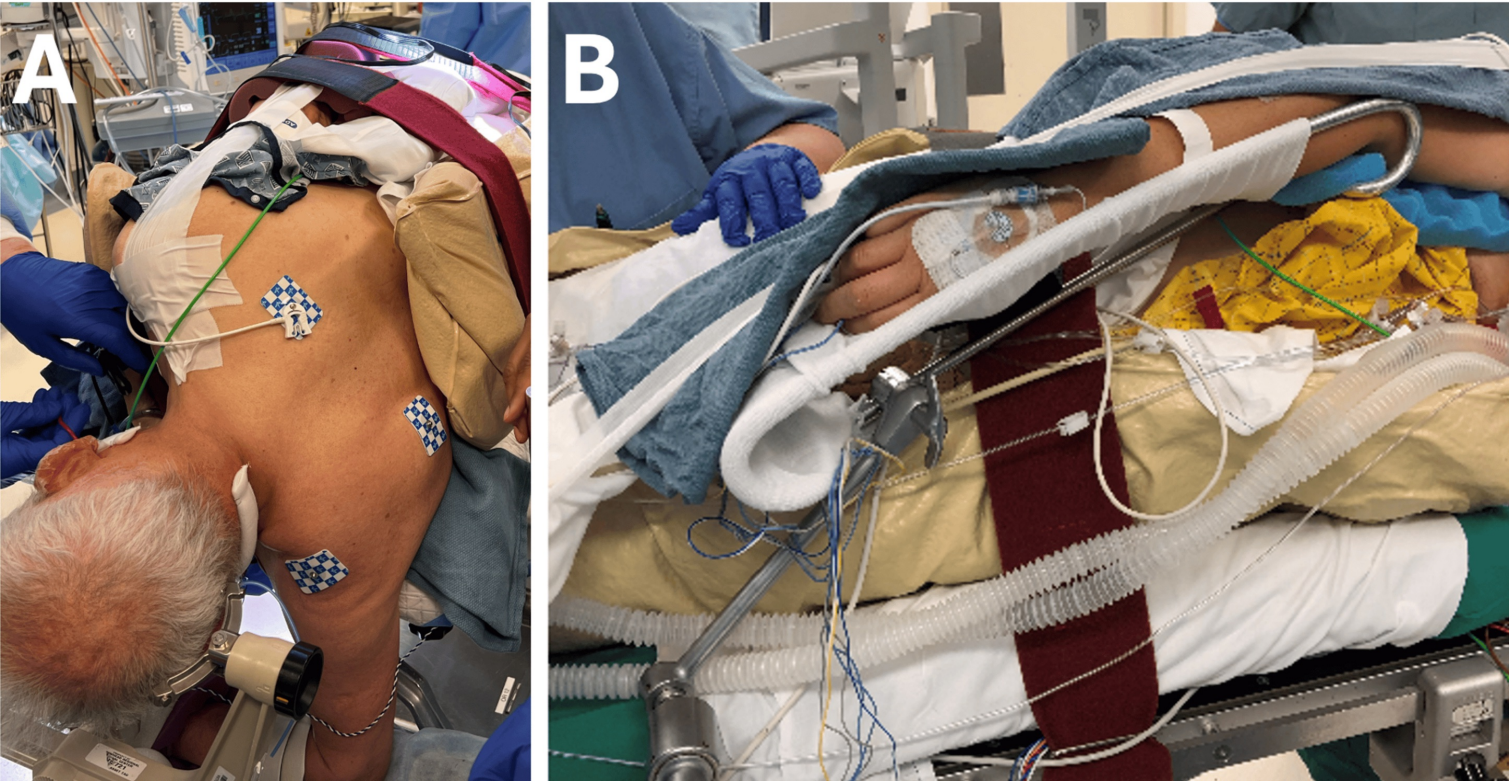
Upper arm positioning A. A pillow is wedged in the axilla. Tape is used to pull the shoulder anteriorly and caudally, out of the surgeons’ working space. B. The Krause-style arm support is attached to the operating table side rail facing the patient and maintains the patient's arm at the same height as their ipsilateral hip [1].

Upper arm: It is supported by a pillow wedged in the axilla. Tape is then applied to the shoulder and attached to the foot of the bed frame to rotate the arm anteriorly with gentle caudal traction, as shown in Figure 6A. This maneuver provides the surgeon with a greater working zone. While gentle traction is desired, care is taken to avoid creating too much tension, as this can overstretch the brachial plexus, leading to nerve palsies [2]. An alternative option that we use for obese patients is the Krause-style arm support [1], shown in Figure 6B. This arm support keeps the thorax open, which helps optimize respiratory mechanics and ventilation, making it ideal for obese patients [1].

Step 7: Final Adjustments and Endotracheal Tube (ETT) Considerations

Neuromonitoring leads are now placed. A Bair Hugger is placed over the patient, and the three Velcro straps are then secured over the Bair Hugger. A portion of the ETT neck strap courses through the planned surgical site and is therefore cut and re-secured to the patient's neck using staples. At times, securing the ETT to one arm of the Mayfield may also be prudent to avoid inadvertent and invisible (under the drapes) ETT slippage due to staple loosening.

**Figure 7:**
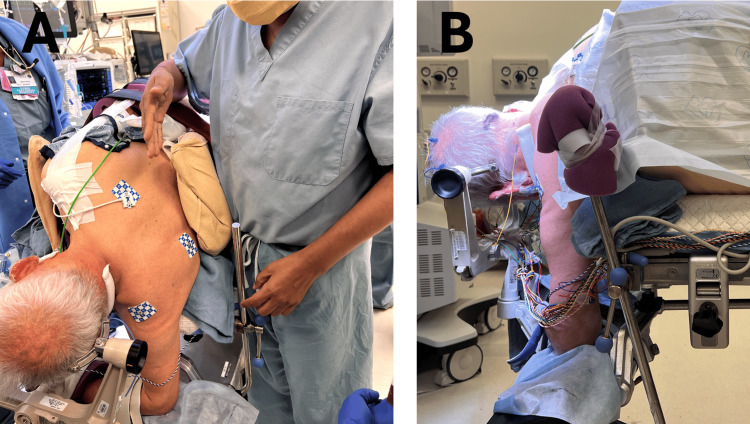
Final adjustments A. The upper end of the lower arm support bar would penetrate the physician's torso when standing in the ideal operating position. B. Foam padding is placed around the upper end of the support bar to cushion the impact and allow the surgeon to operate comfortably.

As shown in Figure 7A, the upper end of the lower arm support bar may inhibit the surgeon from comfortably accessing their desired position. Attempts should be made to avoid this while initially positioning the lower arm. However, if this is unavoidable, foam padding can be placed around the upper end of the support bar to cushion the impact as shown in Figure 7B.

**Figure 8:**
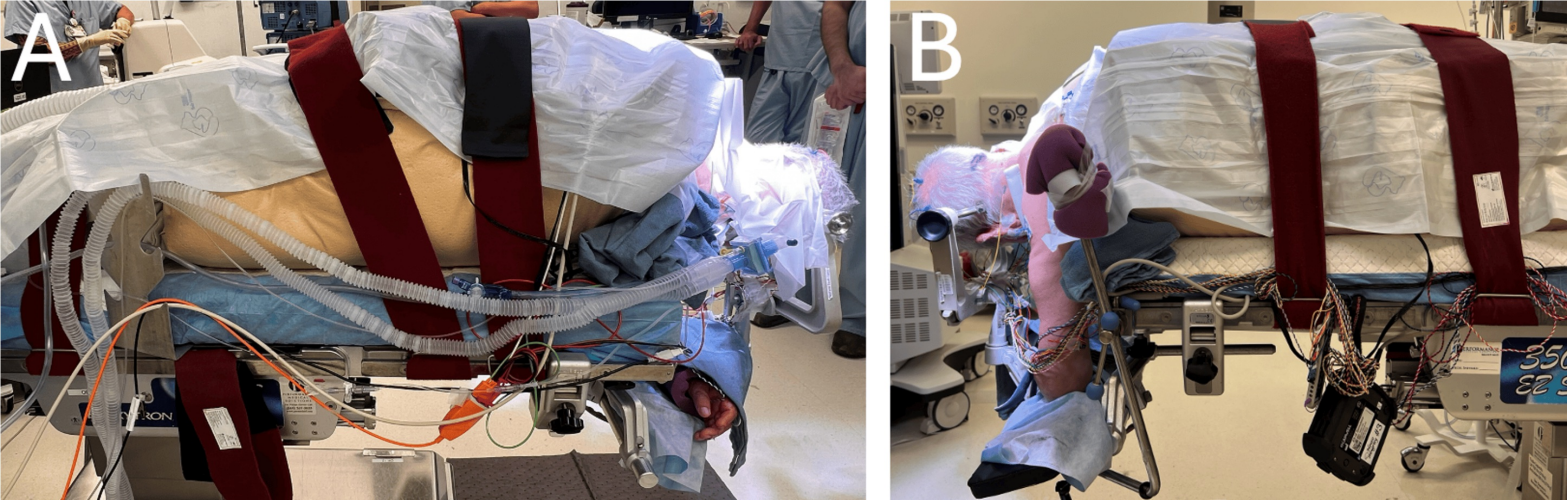
Final positioning A. An anterior-to-posterior (AP) view of the final positioning is demonstrated.  B. A posterior-to-anterior (PA) view of the final positioning is demonstrated.

Tilt the operating table into a small degree of reverse Trendelenburg to minimize venous congestion [2]. Adjust the lateral tilt as needed, ensuring that the patient remains stable and that the body is properly supported [1]. A final safety inspection is then conducted by all team members to ensure that all equipment is correctly positioned and that the patient is secure without their skin contacting hard surfaces. Figure 8 shows the final patient positioning in the park bench position using the Arrowhead technique.

Part II: the Arrowhead technique, microvascular decompression of trigeminal neuralgia 

Skin Incision and Surface Landmarks

**Figure 9:**
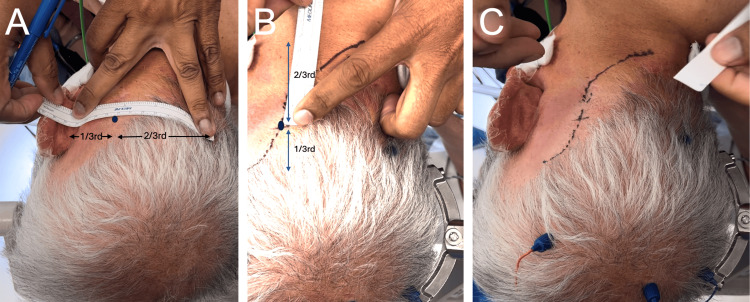
Creating our skin incision A. Measure 2/3^rds^ the distance from the inion to the back of the ear (at the level of the zygoma) and mark this point (represented by the blue dot). In this patient, the distance from the inion to the back of the ear measured 12 cm, meaning this point is 8 cm lateral to the inion along this path. This point represents where the incision will cross the horizontal axis. B. The incision is then drawn using these measurements. Meaning, that 1/3^rd^ (4 cm) of the incision extends above point 1, and 2/3^rds^ (8 cm) of the incision extends below point 1. Hence the mnemonic “2/3^rds^, 1/3^rd^,1/3^rd^, 2/3^rds^”. C. Here we demonstrate the final skin incision.

We use an “S” shaped incision, starting from above the ear and extending inferiorly and medially to the approximate level of the spinous process of C2. The technique we use to create our skin incision can be remembered by the mnemonic “2/3^rds^, 1/3^rd^, 1/3^rd^, 2/3^rds^.” This is explained further in Figure 9.

**Figure 10:**
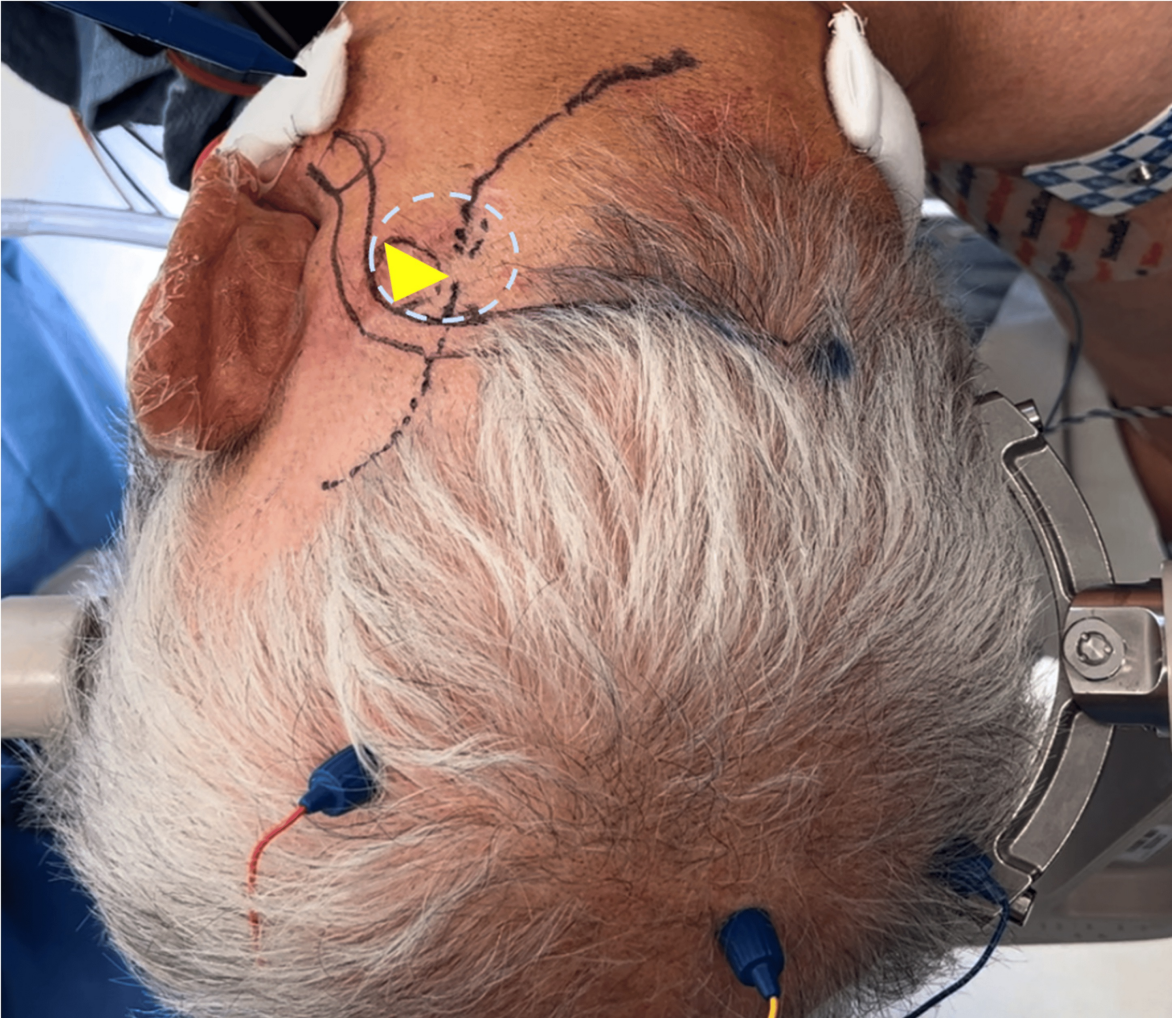
Surface landmarks The tentorium lies deep in the transverse sinus, while the petrous bone lies deep in the sigmoid sinus. The point at which the tentorium and petrous apex conjoin triangulates the working corridor. This is represented by the yellow triangle. The dotted blue circle represents our planned craniectomy outline.

Anatomical surface landmarks are used to estimate the location of the asterion, where we anticipate the transverse and sigmoid sinus junctions to be. A horizontal line is first drawn from the Inion to the root of the zygoma (inion-zygomatic line). The inion-zygomatic line medial to the digastric groove of the mastoid process approximates the transverse sinus. A vertical line is then drawn from the mastoid groove, and the point where these two lines intersect marks the transverse sigmoid junction. Notice our planned “lazy S”-shaped incision courses through the lateral aspect of the transverse sinus, just prior to it becoming the transverse sigmoid junction (Figure 10). The inferior aspect of this incision is aimed towards the midline, away from the putative vertebral artery above the C1 arch. Our working corridor is represented by the yellow triangle in Figure 10, which reflects the intradural triangle, or “angle, of the CPA, which is the primary trajectory for the MVD of TN-ie, the angle between the petrous bone laterally and the tentorium superiorly. During the inferior aspect of the skin opening, careful attention is paid to palpate and auscultate (with micro-Doppler) for any errant course of the vertebral artery on the superior arch of C1. Our planned craniectomy outline is represented by the light blue dotted circle in Figure 10. Now that these landmarks have been drawn in, notice how our planned craniectomy outline is centered at the midway point between both ends of our incision.

A craniectomy is then performed adjacent to the transverse sigmoid junction. We recognize this operation is typically performed through a small (approximately 2 cm diameter) craniectomy; however, we prefer to perform a larger craniectomy (approximately 3 cm in diameter), because in our hands we find it easier to avoid unnecessary retraction on the cerebellum throughout the operation. We then perform a Y-shaped dural opening, with the upper right wing of the Y extending towards the transverse-sigmoid junction and the inferior limb of the Y aimed at the inferior aspect of the craniectomy. The inferior limb of the Y is approximately one cm in length and is opened first; a narrow brain ribbon is passed through the dural opening inferiorly, and gentle pressure is applied until the cisterna magna arachnoid is disrupted and cerebrospinal fluid (CSF) egress is visualized. After a good amount of CSF is released, and the cerebellum is pulsating well and appears decompressed, the other two limbs of the Y-shaped dural opening are completed. This is followed by tack-up sutures to gently reflect the transverse and sigmoid sinus away from our operative corridor.

Intradural Portion of the Procedure

**Figure 11:**
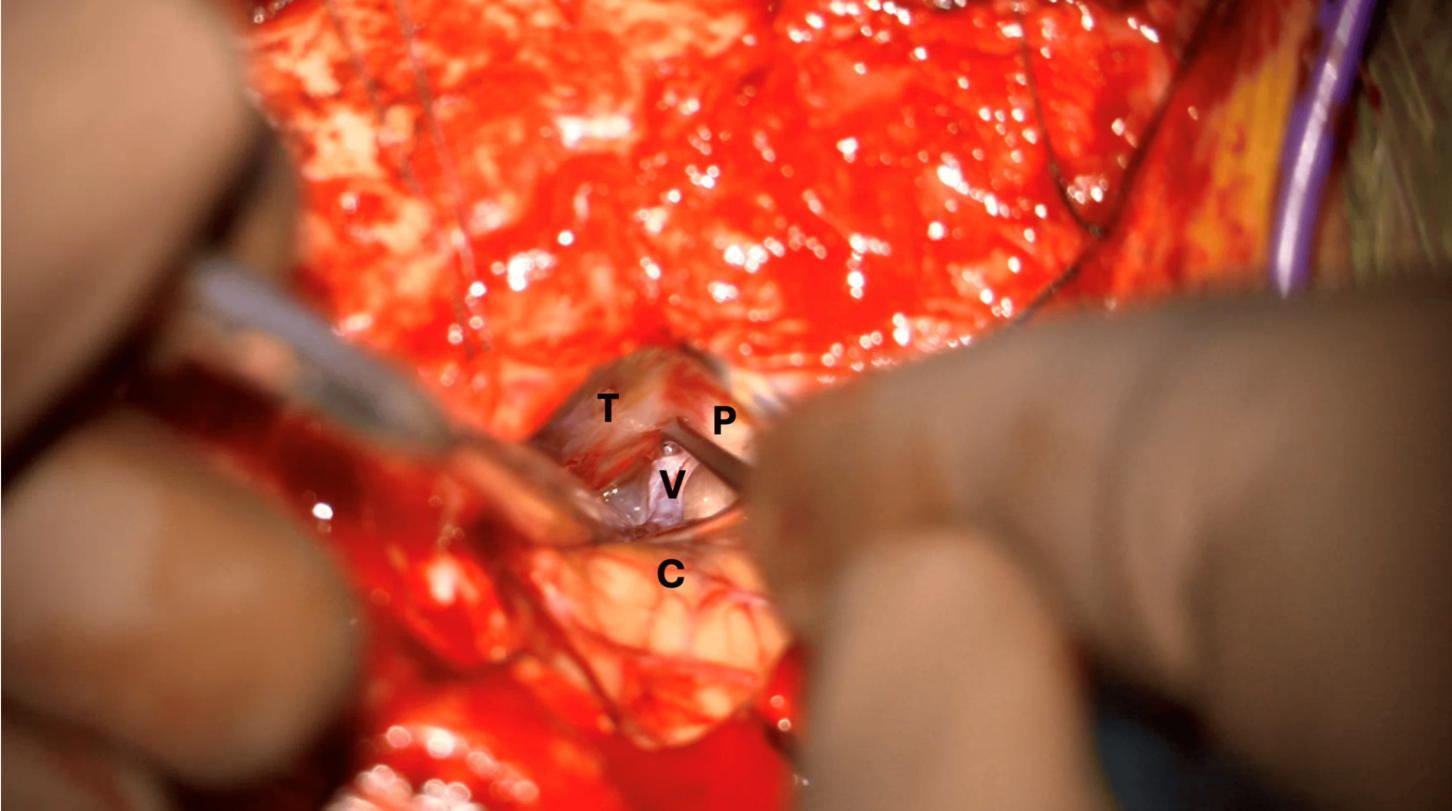
Superior lateral CPA triangle (right side) The superior lateral cerebellopontine angle (CPA) triangle is the working corridor for this operation. The borders of this triangle include the petrous portion of the temporal bone (P), which forms the upper right edge of the triangle. The tentorium (T) forms the upper left edge of the triangle. The cerebellum (C) is the base of the triangle. The superior petrosal vein (V) is seen in the center of this triangle, extending from the base to the apex (the junction where the tentorium and petrous temporal bone meet), and serves as a key landmark. Image key: P denotes the petrous portion of the temporal bone; T denotes the tentorium; C denotes the cerebellum; V denotes the petrosal vein

The superior lateral portion of the CPA is then accessed, and key anatomical landmarks are identified as shown in Figure 11.

**Figure 12:**
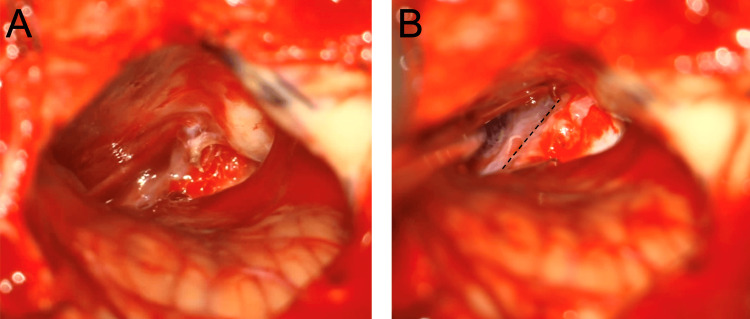
Working triangle after the superior petrosal vein is sacrificed A. View directly after the superior petrosal vein is sacrificed. B. The arachnoid dissection then continues in the direction of the dotted lines.

The trigeminal nerve lies deep in the superior petrosal vein (SPV); therefore, the SPV is sacrificed in a controlled manner. We elect to take the SPV at the beginning of the case because inadvertent tears can result in retraction of the vein into the tentorium, which can become very challenging to control. The working triangle after the SPV is sacrificed is shown in Figure 12. The primary trajectory for the MVD for trigeminal neuralgia is towards the apex of the CP angle, comprised of the petrous bone on the lateral side and the tentorium on the medial side.

**Figure 13:**
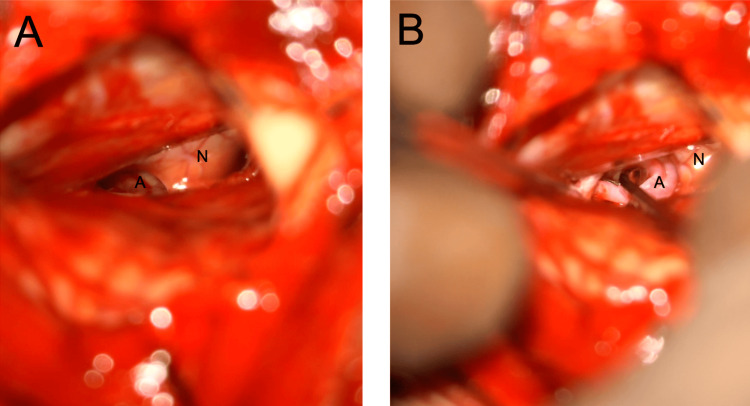
The SCA seen compressing the trigeminal nerve SCA: superior cerebellar artery Image key: A denotes the SCA; N denotes the trigeminal nerve

Arachnoid dissection continues, and the superior cerebellar artery (SCA) compressing the trigeminal nerve comes into view, which is shown in Figure 13. Although not pictured, cranial nerve (CN) V extends forward toward Meckel’s cave.

**Figure 14:**
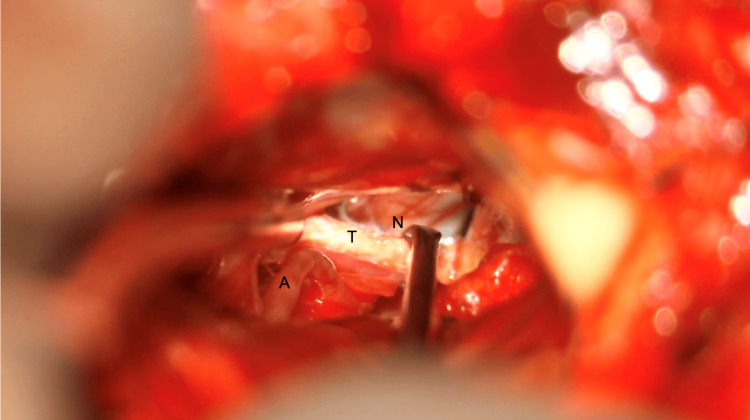
Teflon placement between the trigeminal nerve and SCA SCA: superior cerebellar artery Image key: N denotes the trigeminal nerve; T denotes Teflon; A denotes the superior cerebellar artery In addition to decompression at the root entry zone, any other areas of vascular compression along the nerve cisternal segment should also be addressed. Tisseel is then used to prevent the Teflon from dislodging (not pictured).

Teflon is then inserted between the TN and the offending vessel (Figure 14) to achieve complete decompression at the root entry zone. The circumference of the CN V root entry zone is inspected to ensure that there is no second compression by any aberrant artery or vein.

Closure

After meticulous hemostasis is achieved, attention is turned toward closing the dura. When primary closure is not possible, dural substitutes may be used. Our protocol is the following: DuraGen is placed as an inlay on top of the cerebellum. Dura is then reflected on top of the DuraGen, and the dural edges are approximated with a 4-0 Neurolon suture in a simple interrupted fashion. Another piece of DuraGen is then placed as an onlay, followed by Tisseel. Titanium mesh is used to complete the cranioplasty, ensuring no direct contact between the inner aspect of the scalp and the outer aspect of the dura, which can cause long-lasting pain in some patients. A watertight fascial closure is achieved, and then the skin is closed in a standard fashion. Pressure dressing is applied to the surgical site at the end of the operation to avoid the formation of a pseudo-meningocele. Standard postoperative precautions are taken to avoid a CSF leak.

## Discussion

This technical report provides detailed instructions on the steps used to position a neurosurgical patient in the park bench position for MVD of TN using the Arrowhead technique. Proper patient positioning is the foundation for setting oneself up for a successful operation. Therefore, the entire first section of our report provides detailed nuances on each step of positioning using the Arrowhead technique. While we use the park bench position for this operation, some neurosurgeons prefer to use the supine position with a substantial amount of neck rotation for two main reasons. First, the patient’s shoulder will stay out of the working zone without needing to make further arm adjustments. Secondly, it’s a more familiar position and quicker to set up. 

Our rationale for using the park bench position over the supine position with neck rotation includes the following: A. The park bench position is a more physiologic and natural position for the patient. B. The park bench position avoids putting excess torsion on the patient’s neck, contrary to the supine position. Excess neck rotation can lead to postoperative neck pain and increase the chance of impeding venous return, resulting in venous congestion. C. Should an air embolism occur while operating on a right-sided lesion, the patient is already in the left lateral decubitus position (which is the ideal position for managing venous air embolism). 

The type of surgical incision also varies between neurosurgeons. We use the “lazy S” shaped incision as previously discussed. Other incision types frequently described in the literature include retroauricular C-shaped, linear, and reverse “U” [4, 5]. Our rationale behind the “lazy S” shaped incision is that this incision type provides access to a larger surgical field, and should the craniectomy need to be extended in the event of an unexpected intraoperative complication, it can be done so much more rapidly. Also, we feel that placing the caudal aspect of the incision at the midline provides an additional safety barrier against the rare complication of injuring the vertebral artery during the opening portion of the procedure. 

Finally, there are different intradural approaches used in performing MVD for TN. As previously described, we aim directly for the junction of the petrous bone and tentorium. However, some authors advocate for first identifying the CN VII/VIII complex and opening its cistern prior to proceeding superiorly and anteriorly to the trigeminal nerve [6]. Their rationale is that by first opening this cistern, the cerebellum will become more relaxed, creating a larger working corridor while minimizing retraction on the cerebellum. While this method may work for some surgeons, we prefer the Arrowhead technique for the following reasons: First, it still provides a large enough working corridor to complete the operation without retractors. Secondly, the risk of hearing loss in MVD for TN is between 5.58% and 13.7% [7], thus we prefer to avoid the CN VII/VIII complex altogether. 

In summary, this technical report provides detailed instructions on the steps involved with the park bench position for MVD for TN using the Arrowhead technique. This should serve as a positioning guide for neurosurgery residents to follow. Additionally, it provides nuances and technical insight on other key aspects of performing a MVD. Finally, this article exemplifies the importance of patient positioning for reaching the correct CPA operative corridor. 

## Conclusions

Proper execution of the park bench position is critical for successfully accessing the CPA and is a prerequisite to performing a successful MVD for TN. Adherence to the outlined sequence of steps minimizes the risk of complications and ensures that an abundance of time isn’t wasted on patient positioning. The entire positioning process should be a coordinated effort involving the neurosurgery team, anesthesia, and nursing staff to guarantee patient safety, efficient patient positioning, and surgical success. This report describes the Arrowhead technique for the park bench position for MVD of TN. Furthermore, it integrates key technical considerations from the literature and established practices, creating a comprehensive guide to the park bench position for MVD of TN.
